# The Quality of Anti-SARS-CoV-2 T Cell Responses Predicts the Neutralizing Antibody Titer in Convalescent Plasma Donors

**DOI:** 10.3389/fpubh.2022.816848

**Published:** 2022-03-16

**Authors:** Marie Kroemer, Laura Boullerot, Mélanie Ramseyer, Laurie Spehner, Christophe Barisien, Eleonore Gravelin, Adeline Renaudin, Fabrice Cognasse, Pierre Gallian, Olivier Hermine, Karine Lacombe, Pierre Tiberghien, Olivier Adotévi

**Affiliations:** ^1^INSERM, EFS BFC, UMR1098, RIGHT, University of Burgundy Franche-Comte, Besançon, France; ^2^Department of Pharmacy, University Hospital of Besançon, Besançon, France; ^3^INSERM CIC1431, Clinical Investigation Center in Biotherapy, Biomonitoring Plateform, Besançon, France; ^4^Department of Medical Oncology, Biotechnology and Immune-Oncology Platforme, University Hospital of Besançon, Besançon, France; ^5^Établissement Français du Sang Bourgogne Franche-Comté, Besançon, France; ^6^Établissement Français du Sang Auvergne-Rhône-Alpes, Saint-Etienne, France; ^7^SAINBIOSE, INSERM, U1059, University of Lyon, Saint-Etienne, France; ^8^Établissement Français du Sang, La Plaine St-Denis, France; ^9^UMR “Unité des Virus Emergents”, Aix-Marseille Université - IRD 190 - INSERM 1207 - IRBA - EFS - IHU Méditerranée Infection, Marseille, France; ^10^Department of Hematology, Necker Hospital, Paris, France; ^11^Institut Imagine, INSERM UMR1183, Paris University, Paris, France; ^12^Infectious Diseases Department, Saint-Antoine Hospital, AP-HP, Paris, France; ^13^INSERM IPLESP, AP-HP, Sorbonne University, Paris, France; ^14^Department of Medical Oncology, University Hospital of Besançon, Besançon, France

**Keywords:** neutralizing antibodies, COVID-19, COVID-19 serotherapy, T cell, t-lymphocyte

## Abstract

Convalescent plasma therapy has been described as an attractive approach to treat critically ill patients with COVID-19 (Coronavirus disease 2019). The selection of convalescent plasma donors (CPD) is commonly based on neutralizing antibody titer. A better understanding of the quality of immune responses following COVID-19 will enable the optimization of convalescent donors' selection in convalescent plasma programs. The involvement of SARS-CoV-2 specific T cells in the induction and persistence of high affinity anti-SARS-CoV-2 neutralizing antibody is still poorly investigated. In this study, 115 CPD who presented SARS-CoV-2 and who were eligible for plasma donation were included. Comprehensive analysis of T cells together with humoral responses were performed in regards of sex, age and blood group type. High frequency of T cell responses against SARS-CoV-2 related protein such as spike glycoprotein (80.0%), nucleocapsid (NCAP) (70.4%) and membrane protein (VME1) (74.8%) were detected in CPD by e*x vivo* IFN-γ and TNF-α ELISpot assays. Among CPD responders, most exhibited poly-specific T cell responses (75%) defined by the ability to mount responses against at least two SARS-CoV-2 antigens. We found a positive correlation between the magnitude and the poly-specificity of anti-SARS-CoV-2 T cell responses in CPD. Notably, both the magnitude and poly-specificity of SARS-CoV-2 T cell responses were highly correlated with neutralizing antibody titer in CPD. The present study highlights that the poly-specificity and strength of SARS-CoV-2 specific T cell responses predicts neutralizing antibody titer following COVID-19. These observations show the interest to combine T cell assays and antibody titer for the selection of CPD and to a latter extend to assess COVID-19 vaccine efficacy in at-risk patients.

## Introduction

Severe acute respiratory syndrome coronavirus 2 (SARS-CoV-2), the causative agent of COVID-19 (Coronavirus disease 2019), induces symptoms of variable severity; some patients only have mild illness whereas other rapidly become critically ill progressing to an acute respiratory distress syndrome (1). This critical state of the disease supports the immediate relevance for the development of curative and protective therapeutics against SARS-CoV-2 and requires thorough knowledge dealing with the quality of adaptive immune responses induced by the virus.

To date, supportive care for hospitalized patients with COVID-19 including oxygen supply and the use of dexamethasone 6 mg for up to 10 days for patients who received invasive mechanical ventilation are still the cornerstone of the medical care (2). Recently, monoclonal antibody therapies for COVID-19 have proven their ability to decrease viral load and reduce COVID-19 related hospitalization in selected patients when used very early in the disease course (3–5). Yet, no specific curative drug has proven to be effective whatever the clinical feature of the disease (6–9). Apart from monoclonal antibody therapies and convalescent plasma in particular patients who are unable to produce SARS-CoV-2 targeted antibodies, specific drugs for this disease are still being researched and hardly desired (10, 11).

SARS-CoV-2 infection promotes for most of patients specific immune responses of variable frequencies and magnitude depending on targeted viral proteins and disease severity (12, 13). T cells play a critical role in the fight against SARS-CoV-2 virus by promoting effective viral clearance thanks to virus-specific effector T cells and T-dependent antibody production by B cells (14, 15).

Recent findings revealed that the levels of neutralizing antibodies are highly predictive of immune protection from symptomatic SARS-CoV-2 infection (16). Furthermore, the success of COVID-19 vaccination rollout needs to consider a better understanding of neutralizing antibodies durability against SARS-CoV-2. However, the involvement of T cells in the induction and persistence of high affinity anti-SARS-CoV-2 neutralizing antibodies remain still poorly investigate.

We hypothesize that the neutralizing antibody titer from convalescent patients could rely on the quality of T cell responses. To investigate this question, we assessed cellular and humoral immunity among convalescent plasma donors (CPD) eligible for plasma donation after resolution of COVID-19. We found that the neutralizing antibody titer significantly correlated to T cell poly-specificity against SARS-CoV-2-derived viral proteins. These results have significant implications for vaccination, which should take into consideration the quality of vaccine-induced T cell responses *in vivo*. They also highlight the interest to combine both serological and T cell assays for CPD's selection.

## Materials and Methods

### SARS-CoV-2 Convalescent Donors

Convalescent donors were eligible for plasma donation after resolution of COVID-19. Peripheral blood samples from donors were collected at least 15 days after the end of symptoms at the Etablissement Français du Sang (EFS Besançon, France) from April to June 2020. This study is a cross-sectional research. All CPD were enrolled in COVIPLASM study (NCT04345991) after the signature of informed consent and following the EFS guidelines. Peripheral blood mononuclear cells (PBMC) from convalescent donors were isolated from the apheresis ring by density centrifugation on Ficoll gradient (Eurobio). Moreover, PBMC from convalescent donors were cryopreserved in CryoStor (CS10 and CS5) cell preservation media (Sigma-Aldrich) and were conserved in nitrogen for flow cytometry and Enzyme-linked-Immunospot (ELISpot) analysis.

### Synthetic Peptides

Peptides covering SARS-COV-2 spike glycoprotein, membrane protein (VME1) and nucleocapsid (NCAP) were purchased from JPT (Germany). PepMix peptide pools consisted of 15-mer sequences with 11 amino acid overlap, covering the complete sequences of the spike Glycoprotein, NCAP and VME1 proteins. Therefore, immune responses measured by PepMix-derived SARS-CoV-2 proteins represent both CD4 and CD8 T cells.

### Assessment of Spontaneous T Cell Responses Against SARS-CoV-2 by IFN-γ ELISpot Assay

The interferon-γ (IFN-γ) and tumor necrosis factor-α (TNF-α) producing SARS-CoV-2-specific T cells responses were quantified by ELISpot assay. For that, 3 x 10^5^ PBMC per well were cultured in anti-human IFN-γ monoclonal antibody or anti-human TNF-α monoclonal antibody in ELISpot plate with PepMix of SARS-CoV-2 (1 μg of each peptide pool/mL) in X-Vivo 15 medium (Lonza) for 48 h at 37°C, 5% CO_2_. Cells cultured with medium alone or Phorbol-12-myristate-13-acetate/Ionomycin (12.5 ng/mL; 0.5 μg/mL, Sigma-Aldrich) were used as negative and positive controls, respectively. All experiments were conducted in duplicates and each result presented is the mean of the duplicates. The IFN-γ and TNF-α spots were revealed following the manufacturer's instructions (Diaclone). Estimation of specific T cell number was expressed as spot-forming cells (SFC)/10^6^ PBMC and calculated after subtracting negative control values (background). SFC were counted using the C.T.L Immunospot system (Cellular technology limited) and assessed with Immunospot 5.0 analyser software. Responses were considered positive when IFN-γ and TNF-α spot number was ≥10 and ratio 2-fold above background. Only the positive magnitudes of specific immune responses were indicated in this study.

### Flow Cytometry

To evaluate intracellular cytokine production, PBMC were stimulated with PepMix peptide pools of spike glycoprotein, NCAP and VME1 (1 μg of each peptide pool/mL) overnight at 37°C, 5% CO_2_ in the presence of brefeldin A at 10 μg/mL (BD Biosciences). PBMC cultured with medium alone or PMA/IONO were used as negative and positive controls, respectively. After the stimulation, PBMC were washed and stained for 20 min at 4°C in PBS with antibodies following an additional staining with Fixable viability Dye (FvD)-eFluor 506 (eBioscience) for 10 min at 4°C. T cell phenotype was investigated performing surface staining with anti-CD3-Pacific Blue (clone UCHT1; BD), anti-CD4-Alexa Fluor 488 (clone RPA-T4; Biolegend), anti-CD8-PE-Cy7 (clone SK1; Biolegend), antibodies. Then, PBMC were permeabilized and fixed with the Cytofix/Cytoperm kit according to the manufacturer's instructions (BD Biosciences). Intracellular staining was performed using anti-IFN-γ APC (clone 4S.B3; Biolegend), anti- TNF-α APC AF700 (clone IPM2, Beckman Coulter), anti-IL-2 PE (clone MQ1-17H12, Biolegend) antibodies for 30 min at 4°C. Samples were directly acquired on a Cytoflex (Beckman Coulter) and analyzed with Kaluza software.

### Neutralizing Activity of Anti-SARS-CoV-2 Antibodies

Neutralizing antibodies were detected using a virus neutralization test as previously described for other viruses (17). We used VeroE6 cells cultured in 96-well microplates, 100 TCID50 of the SARS-CoV-2 strain BavPat1 (courtesy of Pr. Drosten, Berlin) and serial dilutions of serum (1/20-1/160). Dilutions associated with cytopathic effect (CPE) due to virus growth and no-CPE (neutralization of virus growth by specific antibodies) at day 4 post-infection were considered as negative (no neutralization) and positive (complete neutralization), respectively. The neutralization titer referred to the highest positive dilution of serum. Specimens with a titer ≥40 were considered positive. All samples were tested in twice for all dilution.

### Heat Maps

Heat maps were performed using the online Morpheus software (https://software.broadinstitute.org/morpheus/). Antibody neutralizing titers were defined as negative (<1:40), low (1:40), intermediate (1:80) and high (≥1:160). T cell responses against the spike glycoprotein, NCAP and VME1 were defined as negative (lower than 10 spots), low (10 to 20 spots), intermediate (21 to 300 spots) or high (>300 spots).

### Statistical Analysis

Statistical analyses were carried out with Prism 8 software. All tests were two sided and the level of significance was set at *p* < 0.05 (^*^p < 0.05, ^**^p < 0.01, ^***^p < 0.001, ^****^p < 0.0001). Variables were expressed as median and interquartile range (IQR) or mean (standard deviation) and evaluated with the Mann–Whitney test. Frequency (percentage) was provided for the description of categorical variables. Proportions were compared using the Chi2 test (or Fisher exact test, if appropriate).

## Results

### Convalescent Plasma Donor Cohort

CPD's demographic and clinical characteristics are detailed in Table 1. SARS-CoV-2 infection was confirmed by PCR test after nasopharyngeal swab (*n* = 86) or positive serology (*n* = 111). CPD eligible for plasma donation were enrolled at least 32 days (10–60) after resolution of COVID-19. None of them were hospitalized because of the disease. The median age was 37 years (20–65), and 71 (61.7%) were male. Blood group were respectively O (41.7%) A (36.5%), B (12.2%) and AB (9.6%).

**Table 1:** Characteristics of SARS-CoV-2 convalescent plasma donors.

	**COVIPLASM cohort**
Sex (*n* = 115)
Women	44 (38.3%)
Men	71 (61,7%)
Age–median (year) and range (*n* = 115)	37 [20–65]
< 30 years	38 (33.0%)
30–50 years	46 (40.0%)
≥ 50 years	31 (27.0%)
ABO blood group (*n* = 115)
O	48 (41.7%)
A	42 (36.5%)
B	14 (12.2%)
AB	11 (9.6%)
Time between COVID-19 infection and samples (days) (*n* = 56)	32 [10–60]
< 30 days	33 (58.9%)
> 30 days	23 (41.1%)
COVID-19 assay	
Positive PCR (*n* = 86)	58 (67.4%)
Positive serology (*n* = 111)	83 (74.8%)

### Poly-Specificity of T Cell Responses Against SARS-CoV-2 Proteins Is Correlated to the Magnitude of Anti-SARS-CoV-2 T Cell Responses

**Figure 1:**
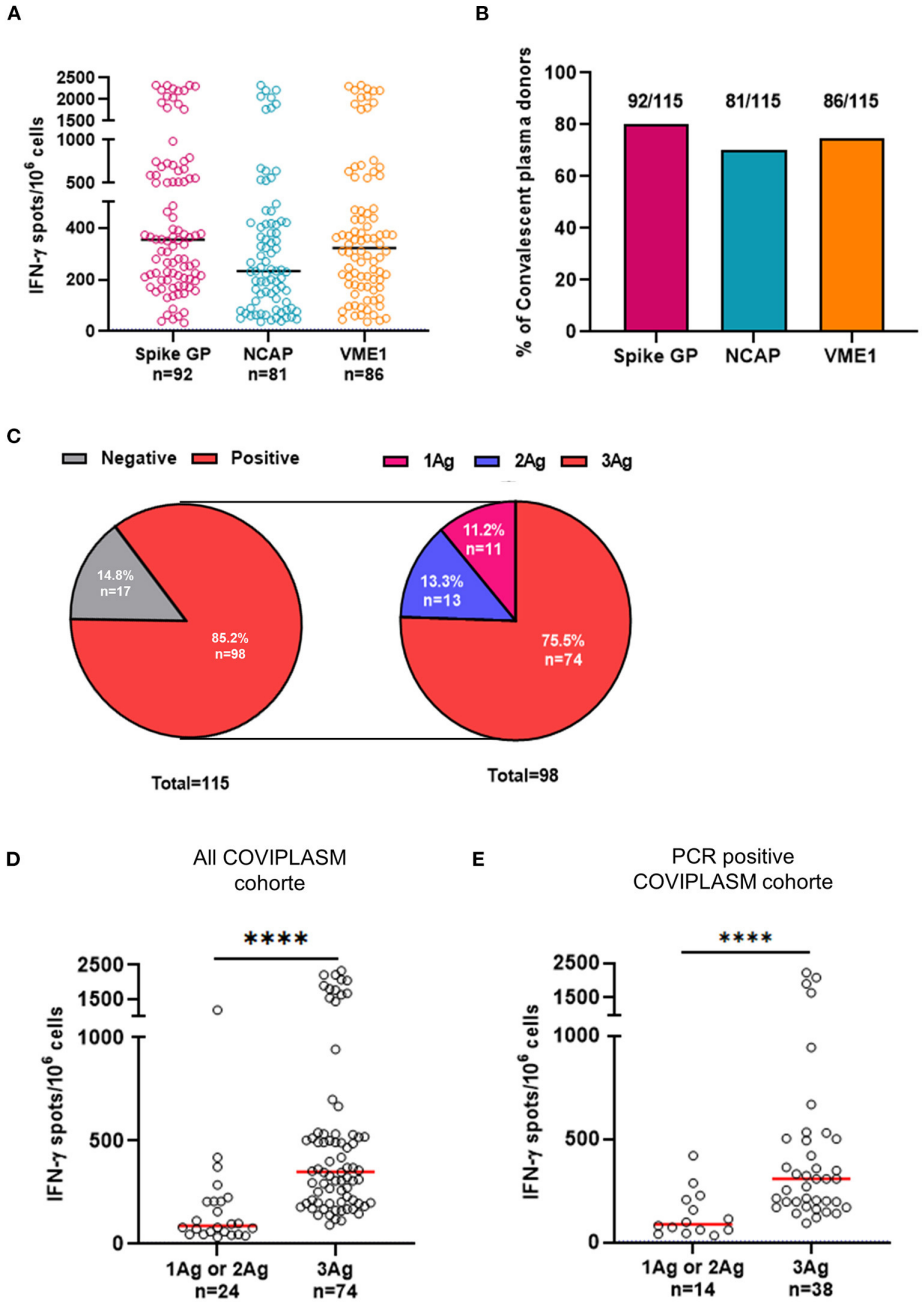
Poly-specificity and magnitude of T cell responses against SARS-CoV-2 derived proteins. **(A)** Magnitude of positive IFN-γ^+^ SARS-CoV-2 specific T cell responses in CPD. **(B)** Frequencies of CPD with T cell responses directed against the SARS-CoV-2. **(C)** Distribution of IFN-γ^+^ SARS-CoV-2 T cell responses (left pie-chart) and distribution of positive IFN-γ^+^ SARS-CoV-2 T cell responses against one, two or three SARS-CoV-2 proteins (right pie-chart). **(D)** Comparison of magnitude of positive responses against one or two SARS-CoV-2 proteins and that of positive responses against the three proteins in all COVIPLASM cohort. **(E)** Comparison of magnitude of positive responses against one or two peptides derived from SARS-CoV-2 proteins and that of positive responses against the three peptides in the PCR positive COVIPLASM cohort. Mann-Whitney test, ^****^P < 0.0001. Ag, antigen; CPD, convalescent plasma donor; NCAP, nucleocapsid; VME1, membrane protein.

To analyze COVID-19 related specific T cell responses, *ex vivo* IFN-γ or TNF-α ELISpot assays were performed to measure effector T cells recognizing viral spike glycoprotein, NCAP and VME1 derived peptides. Anti-SARS-CoV-2 T cell responses in CPD were distributed into three groups of low (10–20 spots), intermediate (21–300 spots), and high responders (>300 spots) (Figure 1A). The median numbers of IFN-γ^+^ specific T cells were 354.5 SFC/3x10^6^ cells [IQR: 203.8–631.3] against spike glycoprotein, 233.0 SFC/3 x 10^6^ cells [IQR: 101.5–419.0] against NCAP and 323.0 SFC/3 x 10^6^ cells [IQR: 178.3–496.0] against VME1 (Figure 1A). As shown in Figure 1B, the frequencies of CPD with T cell responses directed against the SARS-CoV-2 proteins of interest were quite similar. Indeed, 80.0, 70.4, and 74.8% of CPD had T cell responses against spike glycoprotein, NCAP and VME1 respectively (*P* = 0.2443). Similar frequencies and distribution of SARS-CoV-2 specific T cell responses were made by using TNF-α ELISpot assay (Supplementary Figures 1A,B). Similar results were also showed when focusing on the sub-group with COVID-19 PCR positivity assay (Supplementary Figures 1C,D, 2A,B). We observed in most CPD that anti-SARS-CoV-2 specific T cells concurrently produced TNF-α and IFN-γ (Supplementary Figures 1E,F).

Next, we studied the poly-specificity of cellular responses defined as the ability to mount responses against at least two SARS-CoV-2 derived antigens selected here. We found that among immune responders, 75.5% (74/98) displayed T cell responses directed against spike glycoprotein, NCAP and VME1 simultaneously (Figure 1C). Similar results were also showed when focusing on the sub-group with COVID-19 PCR positivity assay (Supplementary Figure 2C). Strikingly, the stronger the magnitude, broad the anti-SARS-CoV-2 T cell response was suggesting a positive link between the quality and the magnitude of anti-SARS-CoV-2 T cell responses in CPD (Figures 1D,E). Of note, no obvious relationship was found between the anti-SARS-CoV-2 T cell response and CPD's demographic and clinical characteristics (Table 2; Supplementary Table 1).

**Table 2:** Distribution of anti-SARS-CoV-2 T cell responses according to convalescent plasma donors' characteristics.

	**Anti-SARS-CoV-2 T cell responses**		
	**Positive** **(*n* = 98)**	**Negative** **(*n* = 17)**	***P* value**
Sex			
Women (*n* = 44)	38 (38.8%)	6 (35.3%)	*p* > 0.99
Men (*n* = 71)	60 (61.2%)	11 (64.7%)	
Age–median (year) and range	37 [20–65]	31 [20–63]	
< 30 years (*n* = 38)	30 (30.6%)	8 (47.1%)	*p* = 0.26
30–50 years (*n* = 46)	40 (40.8%)	6 (35.3%)	*p* = 0.79
≥ 50 years (*n* = 31)	28 (28.6%)	3 (17.6%)	*p* = 0.55
ABO blood group			
O (*n* = 48)	38 (38.8%)	10 (58.8%)	*p* = 0.18
A (*n* = 42)	39 (39.8%)	3 (17.7%)	*p* = 0.10
B (*n* = 14)	10 (10.2%)	4 (23.5%)	*p* = 0.22
AB (*n* = 11)	11 (11.2%)	0 (00.0%)	*p* = 0.36
Time between COVID-19 infection and samples (days)	26 [10–60]	20 [18–37]	
< 30 days (*n* = 33)	28 (58.3%)	5 (62.5%)	*p* > 0.99
> 30 days (*n* = 23)	20 (41.7%)	3 (37.5%)	
Missing	50	9	

*Responses were considered positive when IFN-γ and TNF-α spot number was ≥ 10 and ratio 2-fold above background. When IFN-γ and TNF-α spot number was < 10 or ratio inferior to 2-fold above background, the response the T cell response were considered negative*.

Altogether, these results showed a positive link between the diversity and the level of SARS-CoV-2-specific T cell responses in CPD.

### Both CD4 and CD8 T Cells Are Involved in SARS-CoV-2 Derived Proteins Recognition in CPD and Are Poly-Functional

**Figure 2:**
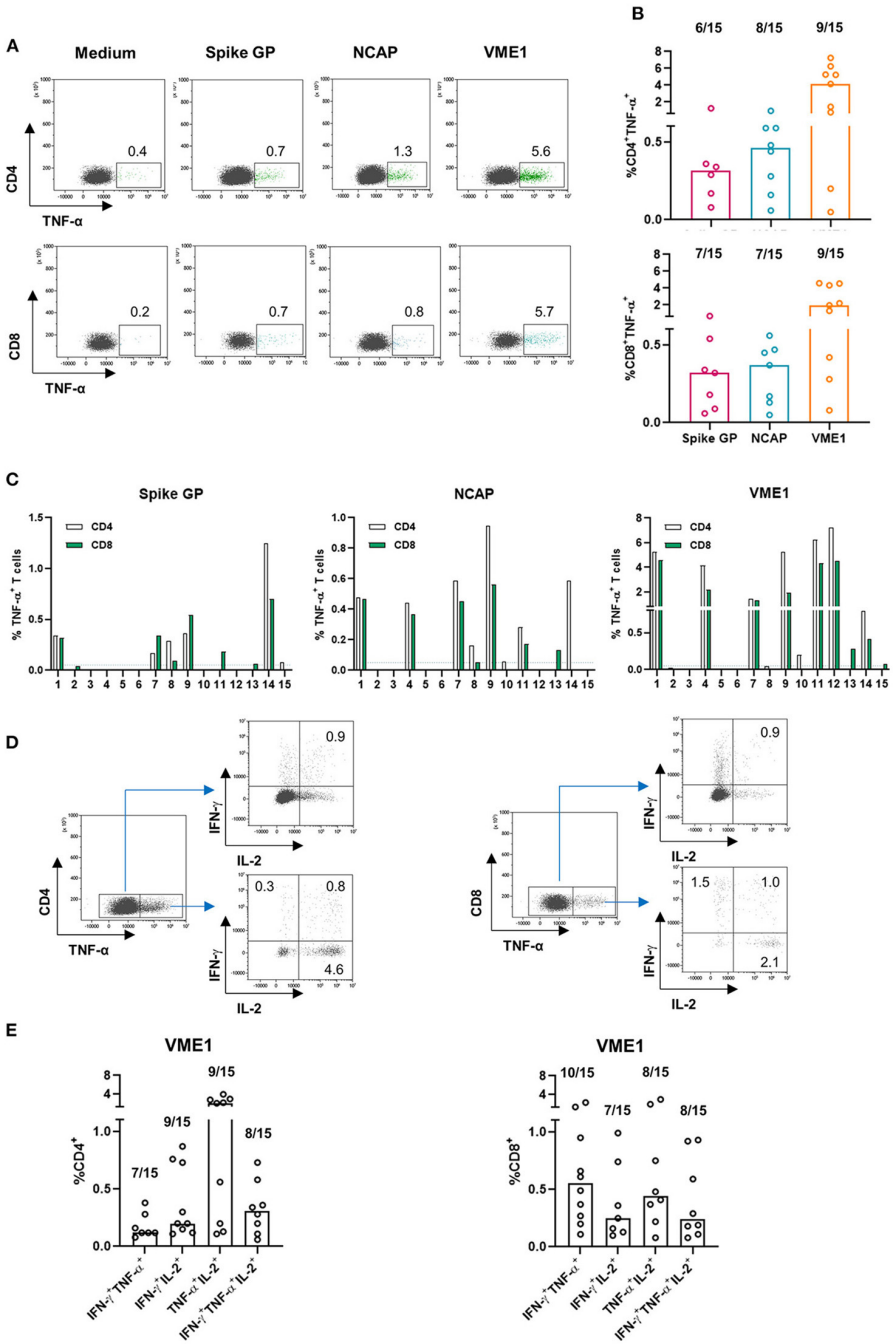
SARS-CoV-2-specific T cells are poly-cytokines producing cells. PBMC were subjected to an overnight stimulation using PepMix peptide pools and analyzed by flow cytometry using intracellular staining for IFN-γ, TNF-α and interleukin-2 (IL-2) cytokines gating on CD4 and CD8 T cell populations (*n* = 15). **(A)** Dot plots are representative of TNF-α secretion by CD4 T cell specific for the spike glycoprotein, NCAP and VME1 protein for one high responder CPD (left panel) and presence of CD4 T cell responses specific for the three SARS-CoV-2 proteins based on TNF-α secretion in 15 CPD (right panel). **(B)** Dot plots are representative of TNF-α secretion by CD8 T cell specific for the spike glycoprotein, NCAP and VME1 proteins for one high responder CPD (left panel) and presence of CD8 T cell responses specific for the three SARS-CoV-2 proteins based on TNF-α secretion in 15 CPD (right panel). **(C)** Presence of CD4 and CD8 T cell responses specific for the three SARS-CoV-2 proteins based on TNF-α secretion among 15 CPD. **(D)** Dot plots are representative of CD4 and CD4 poly-cytokines producing T cells specific for VME1 protein in one high responder CPD. **(E)** Presence of poly-cytokines producing T cells specific for the three SARS-CoV-2 proteins based on IFN-γ, TNF-α and IL-2 secretion in 15 CPD. CPD, convalescent plasma donor; GP, glycoprotein; NCAP, nucleocapsid; VME1, membrane protein.

To further characterize the SARS-CoV-2 specific T cell subsets, we performed intracellular cytokine (IL-2, TNF-α and IFN-γ) staining (ICS) assays in 15 representative CPD (low, intermediate, and high responders based on IFN-γ ELISpot). Representative flow cytometry plots from one high CPD responder were shown in Figure 2A. Results in Figures 2A,B showed that both CD4 and CD8 T cells recognized SARS-CoV-2-derived antigens and produced the same effector cytokines in almost all CPD positive for ICS. We found that most CPD's CD4 and CD8 T cells were directed against VME1 derived epitopes (Figure 2B). We observed a strong correlation between anti-SARS-CoV-CD4 and CD8 T cell responses in CPD. Furthermore, the rate of functional SARS-CoV-2 specific CD8 T cells were lower in CPD without concurrent CD4 T cell induction suggesting a need of CD4 T cell help for optimal anti-SARS-CoV-2 CD8 T cell responses (Figure 2C).

Next, we investigated the poly-functionality of the anti-SARS-CoV-2 T cells detected in CPD. It has been previously reported that virus-specific poly-functional T cells (ability to produce several effector cytokines simultaneously) display greater protective immunity in an infectious context (18, 19). To this end, we focused on cytokine producing CD4 and CD8 T cells specific of VME1 using the gating strategy presented in Figure 2D. As shown in Figure 2E, bi and tri-cytokines producing VME-specific CD4 and CD8 T cells were detected in CPD. Similar observations were made with T cells directed against SARS-CoV-2 derived spike glycoprotein and NCAP (data not shown). Overall, both CD4 and CD8 T cells were involved in SARS-CoV-2 derived protein recognition and results strongly suggest their poly-functionality.

### Neutralizing Antibody Titer in CPD Is Related to the Diversity of SARS-CoV-2-Specific T Cell Responses

**Figure 3:**
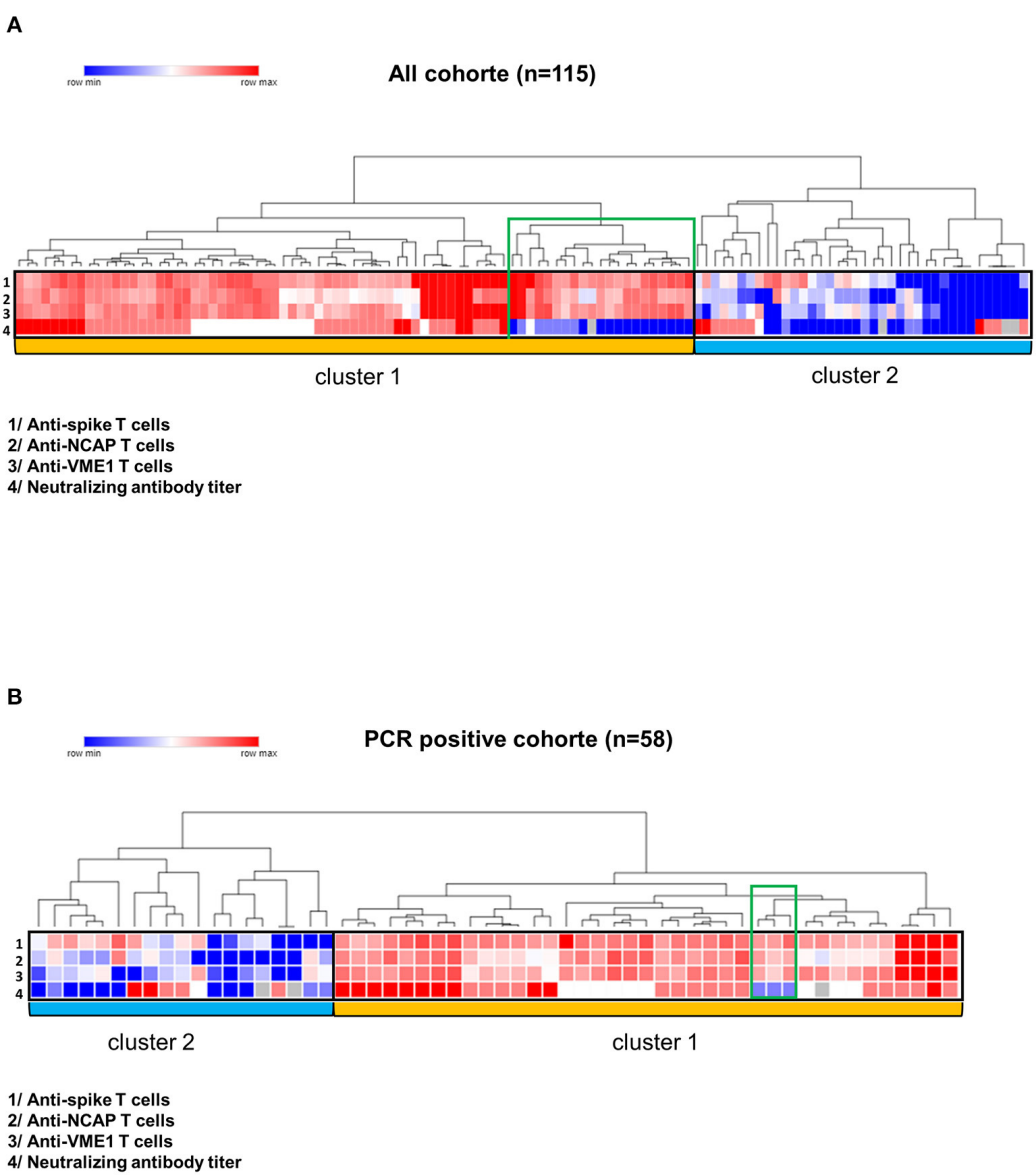
Correlation between T cell responses and neutralizing antibody titers. Heat maps were performed using the online Morpheus software (https://software.broadinstitute.org/morpheus/). Antibody neutralizing titers were defined as negative (<1:40), low (1:40), intermediate (1:80) and high (≥1:160). T cell responses against the spike glycoprotein, NCAP and VME1 were defined as negative (lower than 10 spots), low (10 to 20 spots), intermediate (21 to 300 spots) or high (>300 spots). Unsupervised cluster analysis of immune parameters provided two major clusters (entitled cluster 1 and cluster 2) in all COVIPLASM cohort **(A)** and in the PCR positive COVIPLASM cohort **(B)**. A small group of CPD (indicated in green on heat map) had no neutralizing antibody titer. CPD, convalescent plasma donor; GP, glycoprotein; NCAP, nucleocapsid; VME1, Membrane protein.

Given that a T-B cooperation is required for an efficient production of neutralizing antibody against a pathogen, we therefore investigated the relationship between SARS-CoV-2-specific T cell responses and the level of neutralizing antibody against the spike glycoprotein in COVID-19 controller subjects. Firstly, we performed unsupervised clustering analysis that showed two mains distinct clusters both in all CPD cohort and in the PCR positive cohort (Figures 3A,B). The cluster 1 the most significant in terms of number of CPD, included subjects with poly-specific T cell responses (T cell responses specific against three SARS-CoV-2 proteins) and positive neutralizing antibody titer (titer ≥ 1:40). Thus, cluster 1 was referred as high immune responders. Note that in cluster 1, a small group of CPD (indicated in green on heat map) had no neutralizing antibody titer but T cells directed against the three SARS-CoV-2 antigens of interest. In contrast, the cluster 2 considered as “low immune responders” included CPDs displaying no or low T cell immune response (T cell against one SARS-CoV-2 protein) and low or no neutralizing antibody (Figures 3A,B).

**Figure 4:**
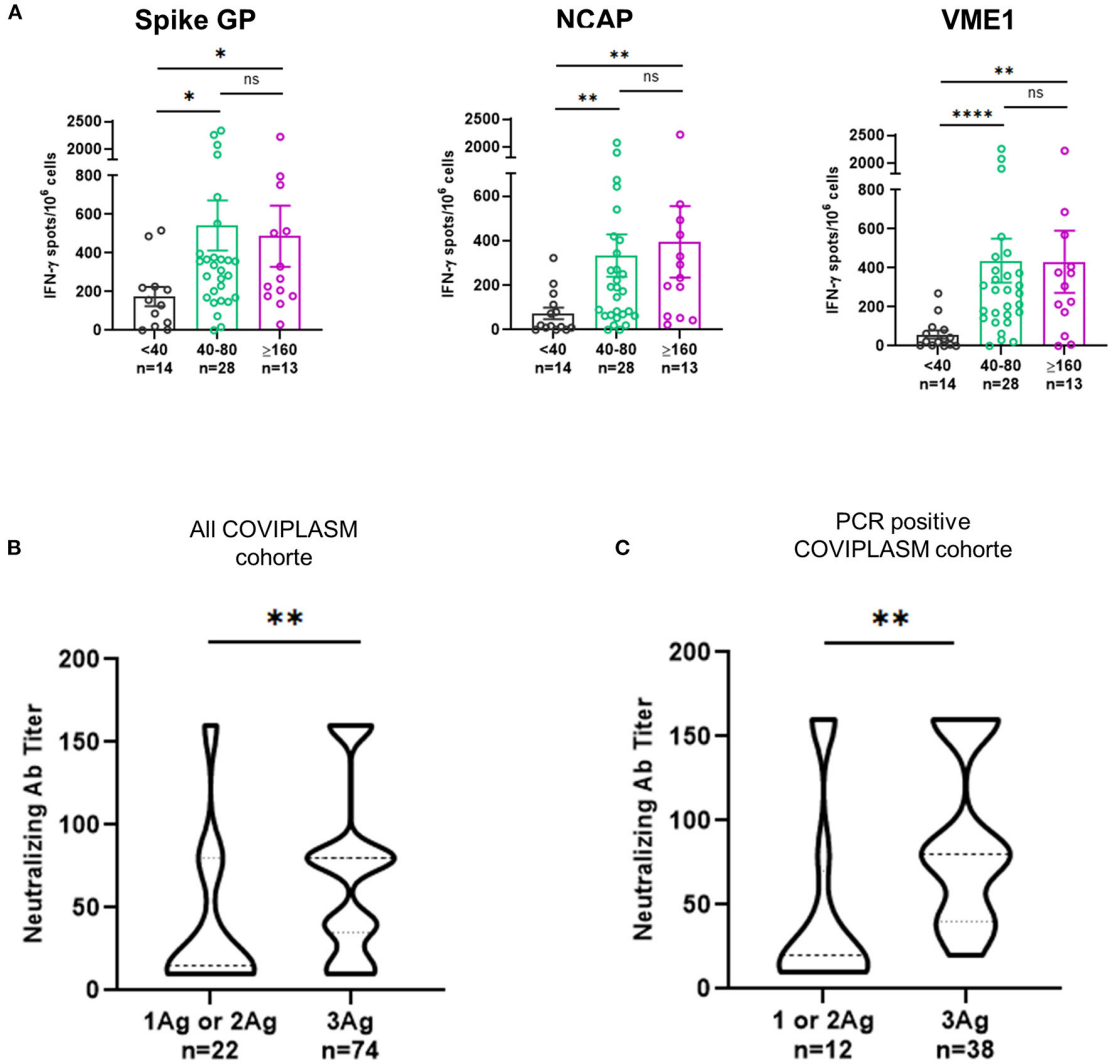
T cell poly-specificity against SARS-CoV-2 derived proteins is highly correlated with neutralizing antibody titer of convalescent plasma donors. **(A)** Magnitude of IFN-γ^+^ SARS-CoV-2-S, VME1 and NCAP T cell responses according to neutralizing antibody titers in PCR positive CPD. **(B)** Neutralizing antibody titers for responses against one or two and three peptides derived SARS-CoV-2 proteins in all CPD. **(C)** Neutralizing antibody titers for responses against one or two and three peptides derived SARS-CoV-2 proteins in PCR positive CPD. Ag, antigen; CPD, convalescent plasma donor; GP, glycoprotein; NCAP, nucleocapsid; VME1, membrane protein. ^*^p < 0.05, ^**^p < 0.01, ^***^p < < 0.001, ^****^p < 0.0001.

These results suggested that the neutralizing antibody titer would be related to the quality (magnitude and diversity) of anti-SARS-CoV-2 cellular response. Hence, we found that CPD with strong T cell responses against spike glycoprotein, NCAP and VME1 had also higher level of neutralizing antibodies (Figure 4A). Furthermore, the titer of neutralizing antibodies was found positively correlated to the poly-specificity of T cells against the SARS-CoV-2 proteins, in all COVIPLASM cohort (*p* = 0.0021) and in the PCR positive one (*p* = 0.0015) (Figures 4B,C). Overall, these observations suggest that the quality of neutralizing antibody response is closely correlated to that of the cellular response against SARS-CoV-2, suggesting the interest to combine these assays to optimize CPD selection and to a latter extend to evaluate vaccination efficacy in at-risk patients.

## Discussion

The present study describes the features of SARS-CoV-2 specific humoral and T cell responses in COVID-19 infection controller subjects eligible for plasma donation after resolution of the infection. Our results demonstrated that CPD exhibited high quality of anti-SARS-CoV-2 T cell responses. Indeed, around 70% of CPD were intermediate to high responders to SARS-CoV-2 using both TNF-α and IFN-γ ELISpot assays. Interestingly, most of CPD developed broad CD4 and CD8 T cell responses directed against three main SARS-CoV-2 derived proteins such as spike glycoprotein, NCAP and VME1. This high diversity of T cell responses found in CPD was also associated with their poly-functionality. Indeed, the anti-SARS-CoV-2 T cells were able to simultaneously produce three effector cytokines such as IFN-γ, TNF-α and IL-2. This supports that high diversity and poly-functional SARS-CoV-2 specific T cell responses contribute to virus clearance in CPD (20). Our results suggest that the presence of SARS-CoV-2-specific CD4 T cells was required for optimal anti-SARS-CoV-2 CD8 T cell induction. This observation is in line with previous observations following vaccination against COVID-19 (21).

An important issue of this study was the close relationship found between the anti-SARS-CoV-2 antibody and T cell responses in CPD. Our results revealed two distinct groups of CPD according to their humoral and cellular responses against SARS-CoV-2. The main group included subjects displaying poly-specific T cell responses against SARS-CoV-2 together with high neutralizing antibody titer. We found that the higher was antibody titer the more diversified were the SARS-CoV-2-specific T cell responses.

The international scope of the COVID-19 crisis supports the interest to investigate parameters that predict the quality of immune responses. High neutralizing antibody titers were recently associated to male sex and older age (22). Here, there was no link between SARS-CoV-2-specific neutralizing antibodies, T cell responses and CPD's demographic and clinical characteristic including ABO blood group. Of note, the distribution of ABO blood group in this cohort was representative of the Caucasian population.

Convalescent plasma therapy has been described as an attractive approach to treat critically ill patients with infectious disease like Ebola, avian influenza (H5N1) and SARS (23–25). The use of convalescent plasma therapy against SARS-CoV-2 virus was suggested early in the COVID-19 pandemic. Nevertheless, results from early clinical trials were disappointing with no evidence of clinical improvement following convalescent plasma therapy in hospitalized patients with severe COVID-19 (26–28). Major biases were suggested such as the limited number of patients with underpowered trials to detect a clinically important difference, the heterogeneity of recipients' clinical characteristics as well as the heterogeneity of convalescent plasma characteristics in terms of antibody titers if measured. Considering the paucity of specific and curative treatment option, the use of convalescent plasma therapy worth to be investigated considering the right time after symptom onset and SARS-CoV-2 specific antibody titer in convalescent plasma. Yet, Libster et al., recently conclude that Early administration of high-titer convalescent plasma against SARS-CoV-2 to mildly ill infected older adults reduced the progression of COVID-19 (29). In the meantime, Joyner et al., performed a retrospective study based on a U.S national registry and conclude that among 3,082 patients hospitalized with COVID-19 who were not receiving mechanical ventilation, transfusion of plasma with higher anti-SARS-CoV-2 IgG antibody titer was associated with a lower risk of death than transfusion of plasma with lower antibody levels (30). All above suggests that the efficacy of convalescent plasma is mainly related to high titer of specific immunoglobulin.

Given that our data demonstrated that high anti-SARS-CoV-2 IgG antibody titers were closely related to the quality of specific T cell responses, we propose to add the analysis of cellular responses for example by ELISpot to the eligibility criteria for plasma donors with therapeutic purposes.

In summary, our data provide new insight regarding the quality of T cell responses following COVID-19. A high T cell response diversity was associated with a high neutralizing antibody titer. This study highlights the interest to consider both cellular and humoral responses for optimal selection of CPD. Our results might also be critical for immune monitoring in the field of COVID-19 vaccination, especially among people at risk of severe COVID-19 infection.

## Data Availability Statement

The original contributions presented in the study are included in the article/Supplementary Material, further inquiries can be directed to the corresponding author.

## Ethics Statement

The studies involving human participants were reviewed and approved by Comité de protection des personnes Ile de France 1 N°IRB/IORG #: IORG0009918. 2020-A00728-31. The patients/participants provided their written informed consent to participate in this study.

## Author Contributions

LB, MR, AR, EG, and LS conducted the experiment. LB, MR, and MK carried out data analysis. OA, PT, PG, and FC designed the research study. MK, OA, PG, FC, OH, KL, and CB discussed the results and wrote and reviewed the manuscript. All authors contributed to the article and approved the submitted version.

## Funding

This article was grant by EFS-UMR 1098.

## Conflict of Interest

The authors declare that the research was conducted in the absence of any commercial or financial relationships that could be construed as a potential conflict of interest.

## Publisher's Note

All claims expressed in this article are solely those of the authors and do not necessarily represent those of their affiliated organizations, or those of the publisher, the editors and the reviewers. Any product that may be evaluated in this article, or claim that may be made by its manufacturer, is not guaranteed or endorsed by the publisher.

## Abbreviations

COVID-19: Coronavirus disease 2019
CPD: convalescent plasma donors
CPE: cytopathic effect due to virus growth
EFS: Etablissement Français du Sang
ELISpot: Enzyme-linked-Immunospot
IFN-γ: interferon-γ
NCAP: nucleocapsid
PBMC: peripheral blood mononuclear cells
PCR: Polymerase Chain Reaction
SARS-CoV-2: severe acute respiratory syndrome coronavirus 2
SFC: spot-forming cells
TNF-α: tumor necrosis factor-α
VME1: membrane protein.
