# Supplementary figures and images for: The Quality of Anti-SARS-CoV-2 T Cell Responses Predicts the Neutralizing Antibody Titer in Convalescent Plasma Donors
Source: Front Public Health. 2022 Mar 16;10:816848. doi: 10.3389/fpubh.2022.816848 (PMC8965758; doi:10.3389/fpubh.2022.816848)

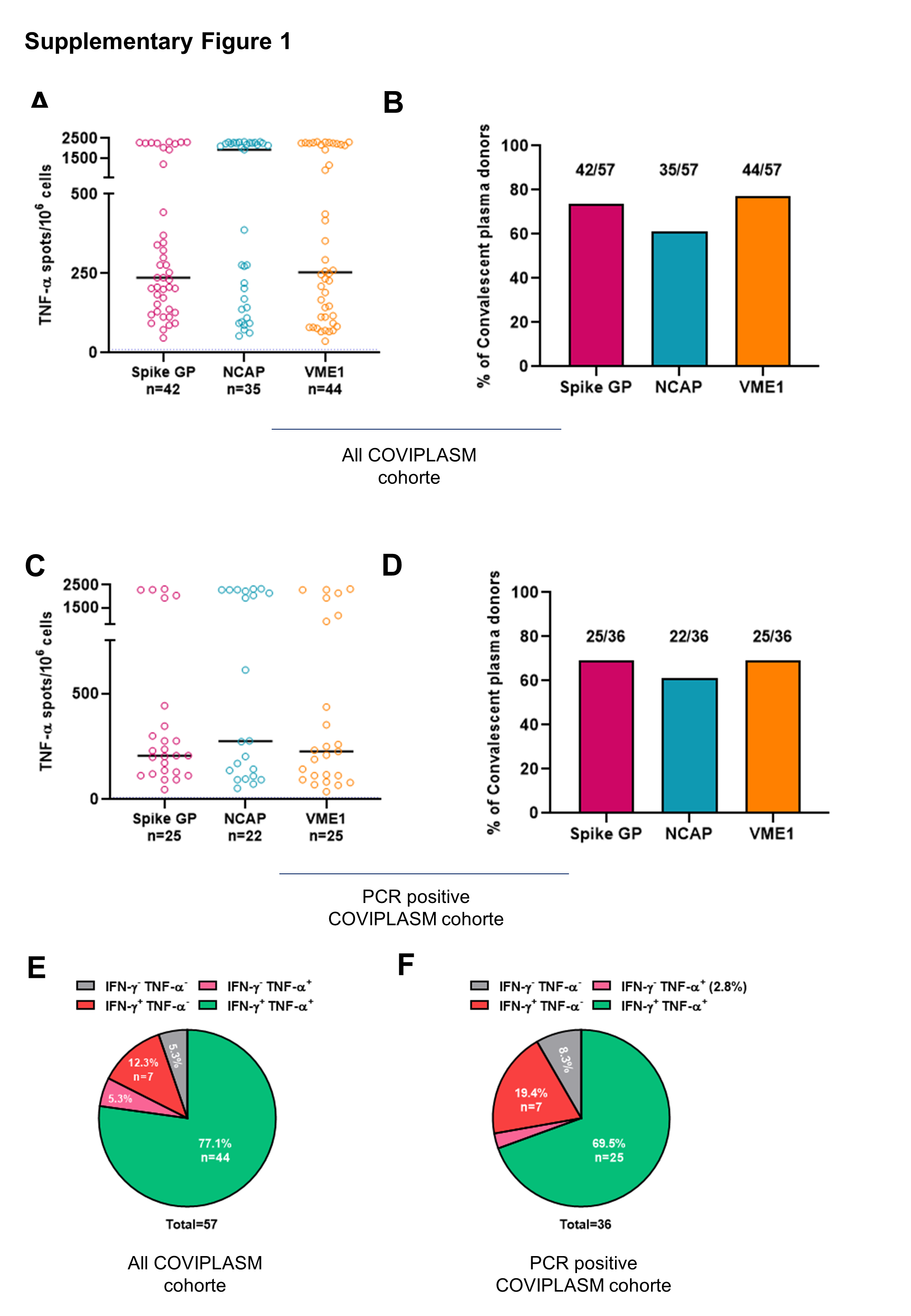

Supplement: Supplementary file 1 [file Image_1.tif]

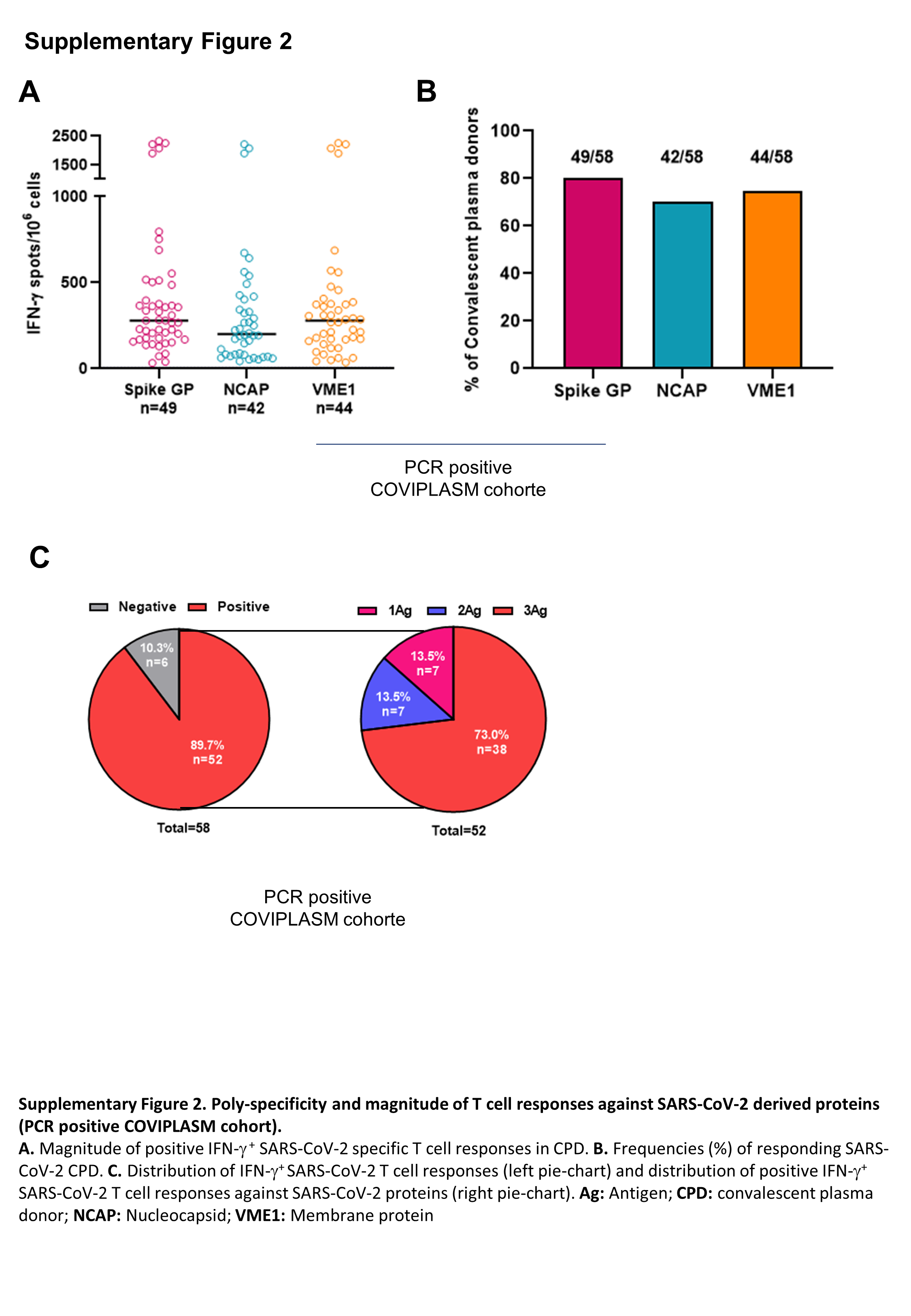

Supplement: Supplementary file 2 [file Image_2.tif]
