# Supplementary material for: The Quality of Anti-SARS-CoV-2 T Cell Responses Predicts the Neutralizing Antibody Titer in Convalescent Plasma Donors
Source: Front Public Health. 2022 Mar 16;10:816848. doi: 10.3389/fpubh.2022.816848 (PMC8965758; doi:10.3389/fpubh.2022.816848)
Supplement: Supplementary file 3 [file Table_1.DOCX]

|  | **Number of IFN-**γ **spots’ median** | | | |
| --- | --- | --- | --- | --- |
|  | **Spike GP** | **NCAP** | **VME1** | **P value** |
| **Sex** |  |  |  |  |
| Women | 367.0 [210.0 – 750.0] | 226.5 [82.3 – 415.8] | 323.5 [167.5 – 598.8] | p=0.26 |
| Men | 310.0 [198.0 – 593.0] | 233.0 [122.0 – 421.0] | 287.0 [187.5 – 462.0] |  |
| **Age** |  |  |  |  |
| < 30 years | 362.0 [179.8 – 543.0] | 236.5 [114.0 – 419.3] | 310.0 [180.0 – 433.0] | p=0.36 |
| 30 – 50 years | 345.0 [204.0 – 662.0] | 233.0 [75.5 – 446.0] | 298.5 [150.0 – 566.8] |  |
| ≥ 50 years | 353.5 [235.0 – 941.8] | 240.0 [108.5 – 458.0] | 377.0 [206.5 – 730.0] |  |
| **ABO blood group** |  |  |  |  |
| O | 343.5 [182.8 – 655.8] | 236.5 [96.8 – 426.0] | 340.0 [173.0 – 470.0] | p=0.20 |
| A | 330.0 [207.8 – 516.0] | 236.5 [84.8 – 382.3] | 298.5 [172.3 – 462.0] |  |
| B | 395.0 [83.5 – 1911.0] | 563.0 [267.0 – 2213.0] | 537.0 [219.3 – 2112.0] |  |
| AB | 548.0 [244.8 – 747.8] | 180.0 [69.0 – 264.0] | 293.0 [155.0 – 527.0] |  |
| **Time between COVID-19 infection and samples (days)** |  |  |  |  |
| < 30 days | 283.0 [198.5 – 531.5] | 197.0 [80.0 – 281.5] | 297.0 [174.8 – 383.5] | p=0.07 |
| > 30 days | 298.5 [207.8 – 511.5] | 246.5 [95.8 – 476.5] | 337.0 [148.5 – 565.0] |  |

**GP:** glycoprotein; **NCAP:** Nucleocapsid; **VME1:** Membrane protein

**Supplementary Table I.** Number of IFN-γ spots’ median according to convalescent plasma donors’ characteristics.
